# Structural analysis of the mylohyoid muscle as a septum dividing the floor of the oral cavity for the purposes of dental implant surgery: variety of muscle attachment positions and ranges of distribution

**DOI:** 10.1186/s40729-023-00513-y

**Published:** 2023-12-08

**Authors:** Taku Noguchi, Sumiharu Morita, Ryu Suzuki, Satoru Matsunaga, Hidetomo Hirouchi, Norio Kasahara, Keisuke Sugahara, Shinichi Abe

**Affiliations:** 1 Department of Anatomy, Tokyo Dental College, 2-9-18 Kandamisaki-cho, Chiyoda-ku, Tokyo, 101-0061 Japan; 2 Oral Health Science Center, Tokyo Dental College, 2-9-18 Kandamisaki-cho, Chiyoda-ku, Tokyo, 101-0061 Japan; 3 Department of Histology & Developmental Biology, Tokyo Dental College, 2-9-18 Kandamisaki-cho, Chiyoda-ku, Tokyo, 101-0061 Japan; 4 Department of Oral Pathobiological Science and Surgery, Tokyo Dental College, 2-9-18 Kandamisaki-cho, Chiyoda-ku, Tokyo, 101-0061 Japan

**Keywords:** Mylohyoid muscle, Sublingual space, Mylohyoid line, Mandibular symphysis, Implant procedural accident

## Abstract

**Objectives:**

The objective was to investigate the details of the attachments of the mylohyoid muscle to the mandible anterior to the hyoid and mylohyoid lines to understand the positional relationship between the sublingual space and the mylohyoid, knowledge that is essential for dental implant surgery in the incisal region, as well as the routes of communication between the sublingual space and other spaces.

**Methods:**

While evaluating the presence or absence of an anterior mylohyoid muscle fiber attachment to the mandible, sublingual gland herniation, spaces between muscle fascicles were also recorded as sites of penetration. The mean muscle thickness in each of these areas was also calculated.

**Results:**

In all specimens, the mylohyoid originated not only from the mylohyoid line but also from the lingual surface of the center of the mandibular body (the mandibular symphysis) below the mental spines. The mylohyoid muscle fascicles were thickest in the posterior region, and further anterior to this, they tended to become thinner. Sublingual gland herniations passing through the mylohyoid were noted in the anterior and central regions, but not in the posterior region. Penetration between the muscle fascicles was most common in the central region, and no such penetration was evident in the posterior region.

**Conclusions:**

These results suggest that the mylohyoid functions only incompletely as a septum, and that routes of communication from the sublingual space to the submandibular space may be present in both the anterior and central muscle fascicles of the mylohyoid. Therefore, bleeding complications during dental implant placement in the anterior mandible can be serious issues. There is a potential for sublingual hematoma that could compromise the airway by pressing the tongue against the soft palate into the pharynx.

## Background

Reported complications associated with dental implant surgery in the mandibular incisal region include severe bleeding and hematoma in the floor of the oral cavity as a result of arterial damage caused by perforation by an instrument that has penetrated through the lingual cortical bone of the mandible [1–5]. The bleeding may easily spread into the loose connective tissue of the floor of the oral cavity and cause airway obstruction [6, 7]. The sublingual and submental arteries that are the source of this bleeding are located in the sublingual space [8, 9]. Understanding the anatomical structure of the sublingual space, and, in particular, the mandibular attachments of the mylohyoid muscle that forms the lower wall of the sublingual space, is thus extremely important for assessing routes of bleeding when an artery is damaged.

The mylohyoid muscle usually originates on the mylohyoid line and terminates on the hyoid body, with the left and right mylohyoids meeting at the midline anterior to the hyoid to form the mylohyoid raphe [10, 11]. Because the mylohyoid is a flat muscle formed of thick muscle fibers enclosed by a strong fascia, it has been described as a septum dividing the floor of the mouth into upper and lower parts. This septum is known to be robust and to block communication between the sublingual and submandibular regions, giving rise to its alternative name of the “oral diaphragm” [10]. The sublingual glands found in the sublingual space have been reported to herniate through the septum formed by the mylohyoid into the submandibular space, and it has been suggested that this may create a route of communication between the sublingual and submandibular spaces, depending on the location of the mylohyoid through which the submandibular glands have penetrated [12–14]. There have been almost no descriptions of the site of attachment to the mandible of the mylohyoid other than on the mylohyoid line, and if there is an area between the incisal and premolar areas in which there is no attachment between muscle and bone, this could provide a route of communication between the upper and lower parts of the floor of the mouth.

The objective of the present study was, therefore, to investigate the details of the mandibular attachments of the mylohyoid muscle anterior to the hyoid and mylohyoid lines to understand the positional relationship between the sublingual space and the mylohyoid, knowledge that is essential for dental implant surgery in the incisal region, as well as the routes of communication between the sublingual space and other spaces.

## Methods

### Specimens

The specimens included the mandibles and the surrounding tissue, including the mylohyoid, collected from 48 Japanese cadavers in the collection of the Department of Anatomy of Tokyo Dental College. The tongue was removed, after which the mylohyoid and its surrounding tissue, including the sublingual glands, were dissected, while the mylohyoid fascia was preserved. This study was conducted with the approval of the Tokyo Dental College Institutional Review Board (IRB No. 11000736, IRB approval No. 781).

### Gross morphological observations and measurements of mylohyoid attachments and muscle fascicles

**Fig. 1:**
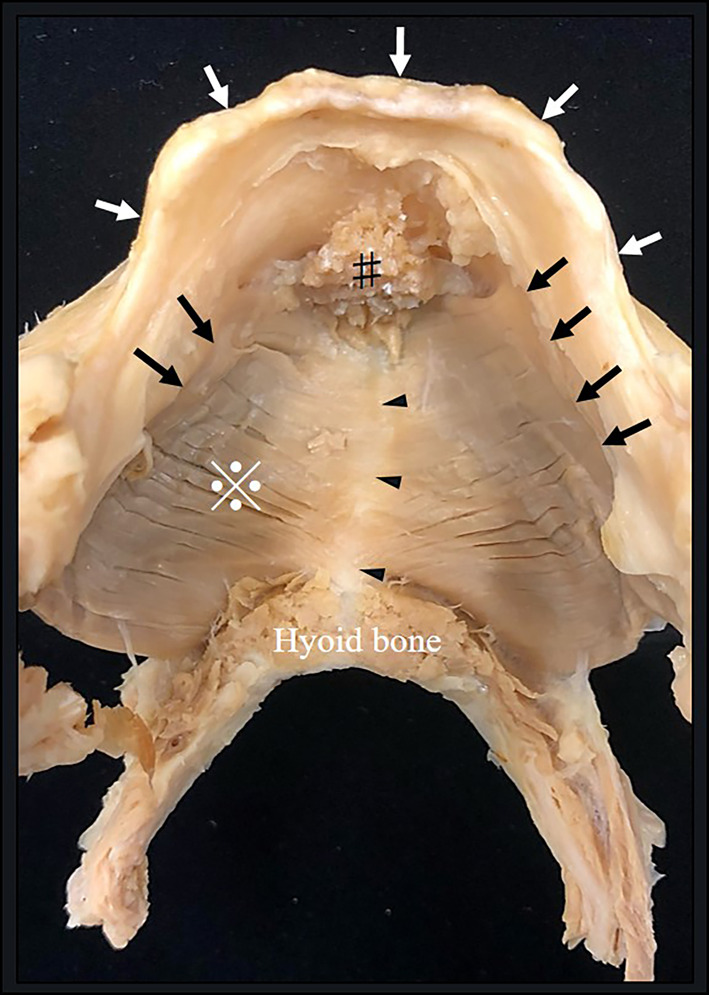
Dissection of the mylohyoid muscle, which attaches to the hyoid and the mandible. ※: mylohyoid muscle. #: geniohyoid muscle (cut). White arrows: alveolar arch. Black arrows: mylohyoid line. Black arrowheads: mylohyoid raphe

**Fig. 2:**
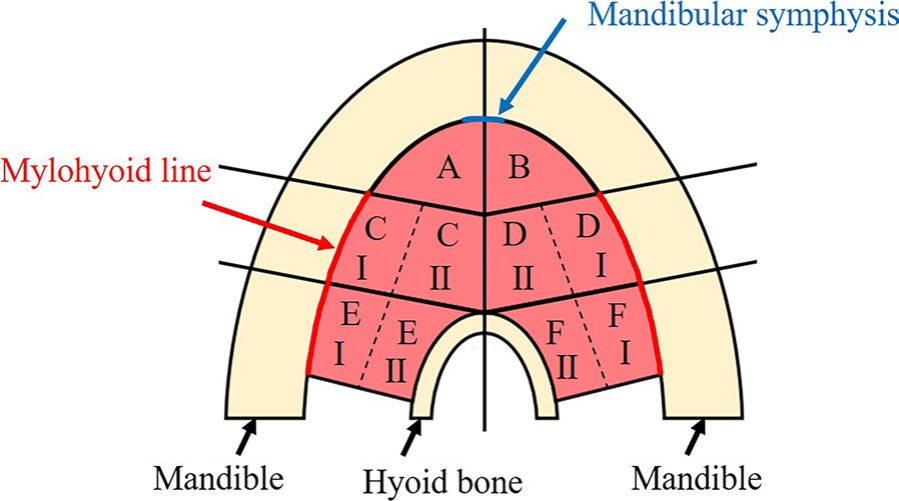
Measurement regions in the mylohyoid. The mylohyoid was divided into six regions of interest, and the muscle fascicles attaching to the hyoid bone and mylohyoid line were defined as posterior (left: **E**, right: **F**), those attaching to the mylohyoid line and mylohyoid raphe as central (left: **C**, right: **D**), and those other than these as anterior (left: **A**, right: **B**). I: side adjoining the mylohyoid line. II: side adjoining the mylohyoid raphe (hyoid bone)

Standard photographs were taken for observations of the dissected specimens (Fig. 1). The mylohyoid was divided into six regions of interest, and the muscle fascicles attaching to the hyoid bone and mylohyoid line were defined as posterior, those attaching to the mylohyoid line and mylohyoid raphe as central, and those other than these as anterior (Fig. 2). While evaluating the presence or absence of an anterior mylohyoid muscle fiber attachment to the mandible, sublingual gland herniation, and the herniated area, a light source was also used to ascertain muscle thickness, and spaces between muscle fascicles into which no sublingual glands were herniated were recorded as sites of penetration. The muscle thickness was also measured at three randomly selected points in each region, and the mean thickness of the regions was calculated.

**Fig. 3:**
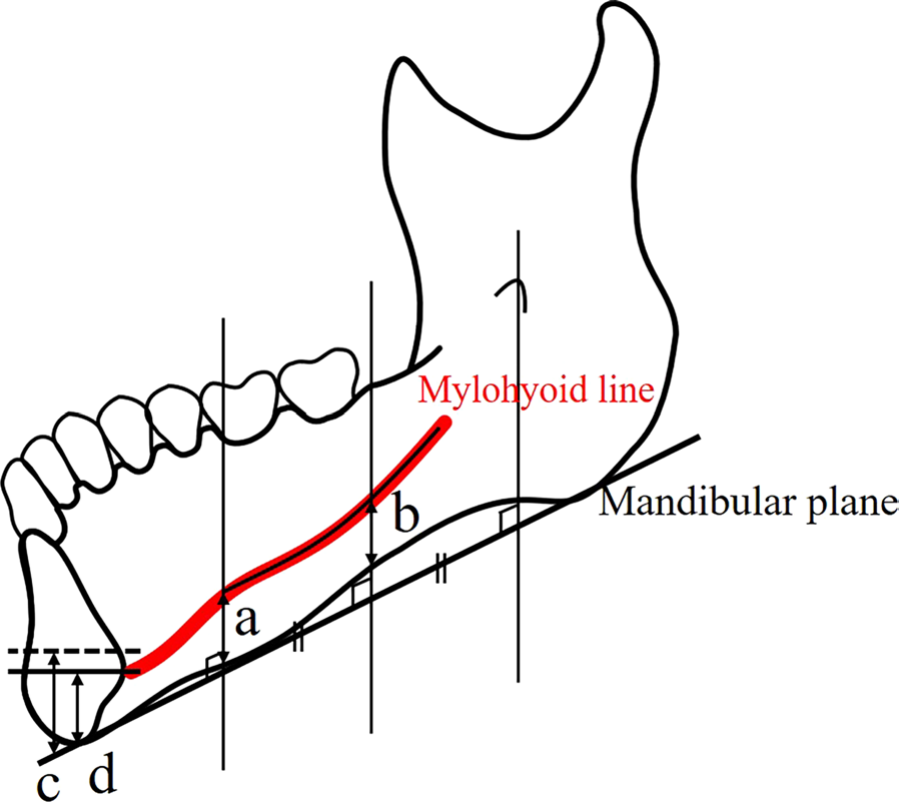
Locations for the measurement of the vertical positions of the mylohyoid attachments. **a** Distance from the anterior margin of the mylohyoid line to the mandibular plane (MFMY) **b** Vertical distance at the midpoint of the distance from the mandibular foramen to the anterior margin of the mylohyoid line (HLMY) **c** Distance between the mandibular plane and the mental spines **d** Vertical distance at the mandibular symphysis

Next, to measure the vertical position of the mylohyoid line, a line joining the anterior margin of the mylohyoid line and the mandibular lingula running parallel to the mandibular plane was divided into two equal parts. The distance *a* (mm) from the anterior margin of the mylohyoid line to the mandibular plane (the distance between the mylohyoid line and the lower margin of the mandible (LMM) at the vertical line of mental foramen (MF): distance between the mylohyoid line and LMM at the vertical line and LMM at the vertical line of MF (MFMY)) and the distance *b* (mm) from the mylohyoid line to the mandibular plane along the vertical line dropped from the point halfway along the line joining the anterior margin of the mylohyoid line and the mandibular lingula (the distance between the mylohyoid line at the point halfway between the lingula and MF and LMM: distance between the mylohyoid line which the half point of the lingula’s notch and MF, and LMM (HLMY)) were then measured (Fig. 3) [15]. At the same time, *c* (mm), the vertical distance from the mandibular plane to the mental spines, and *d* (mm), the distance from the mandibular plane to the anterior attachment of the mylohyoid to the mandible (mandibular symphysis) were also measured.

## Results

### Gross morphological observations of mylohyoid attachments and muscle fascicles

**Fig. 4:**
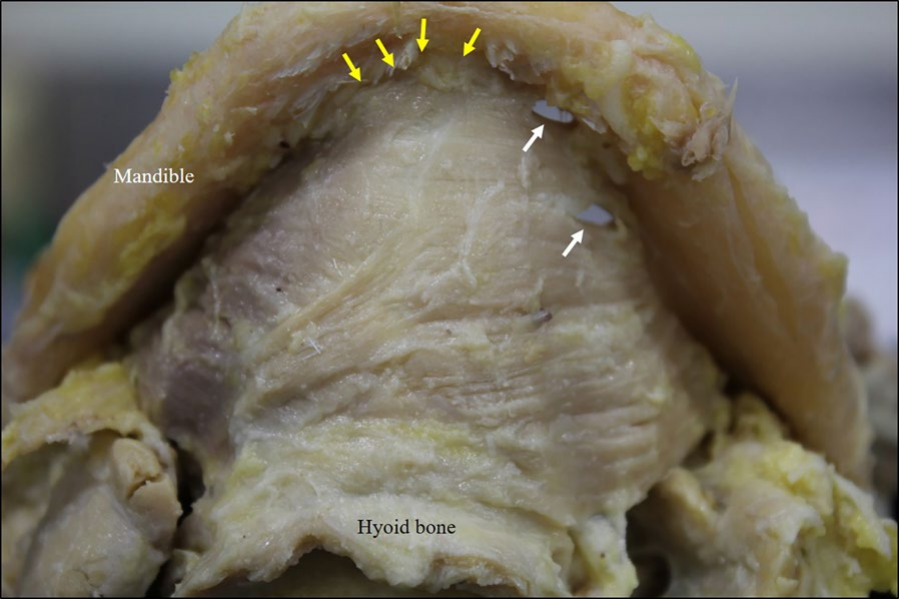
Mylohyoid attachment viewed from below (digastric muscle has been cut off at its origin). The mandibular symphysis from the mylohyoid line is continuous as the origin of the mylohyoid. Two sites of penetration are evident on the left-hand side. a: Mylohyoid line. b: Mandibular symphysis. c: Hyoid bone. d: Mylohyoid raphe. Yellow arrows: mandibular symphysis. White arrows: sites of penetration

**Fig. 5:**
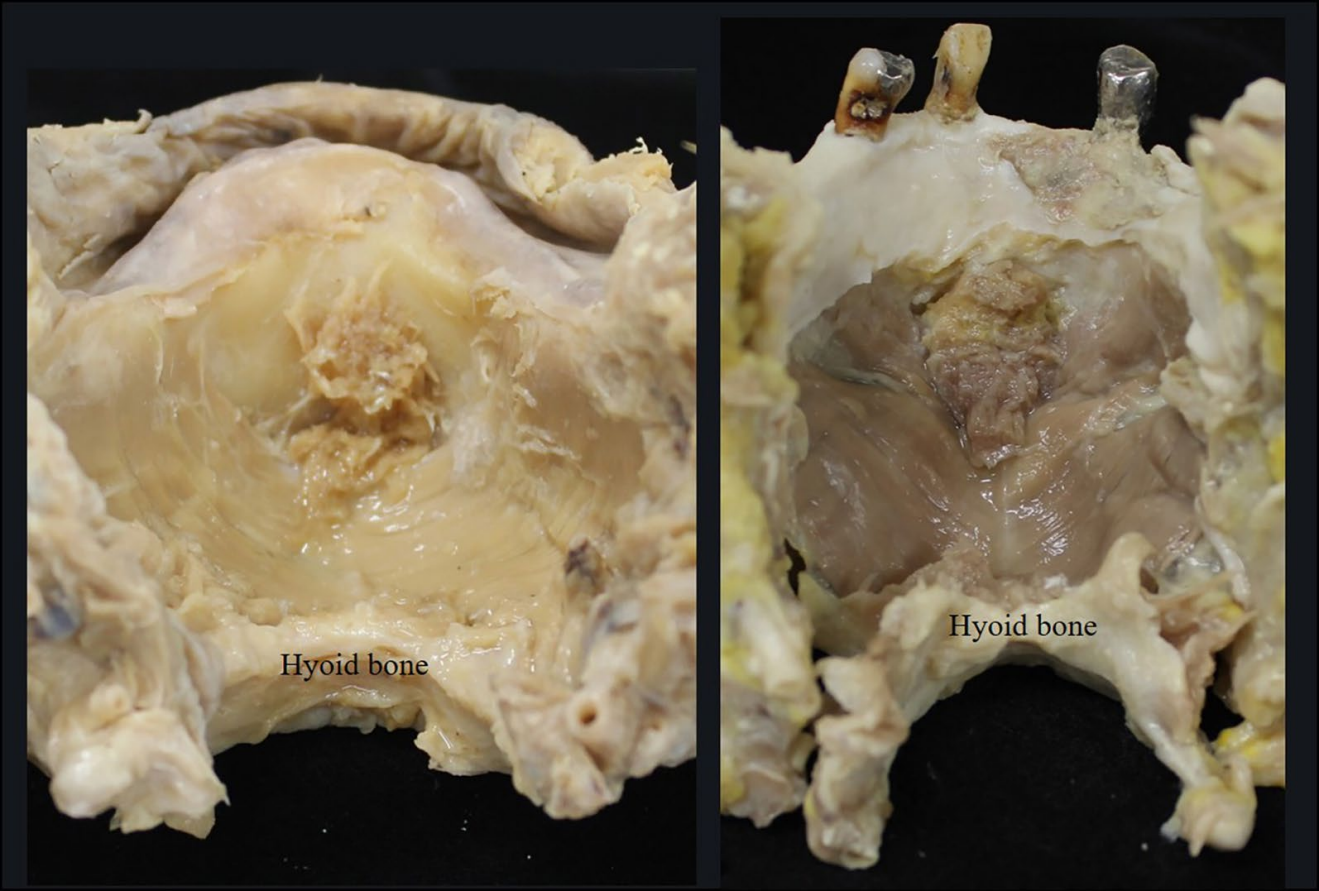
Positional relationship between the mylohyoid raphe and the hyoid bone. (Left: normal type. Right: herniated type) The left and right mylohyoids form an inverted-roof shape with the mylohyoid raphe at its crest. The mylohyoid, which attaches from the mandibular symphysis and the mylohyoid line to the mylohyoid raphe and the hyoid bone, consists of muscle fascicles regularly arrayed to form a sheet, and it divides the sublingual and submandibular spaces from each other.

**Fig. 6:**
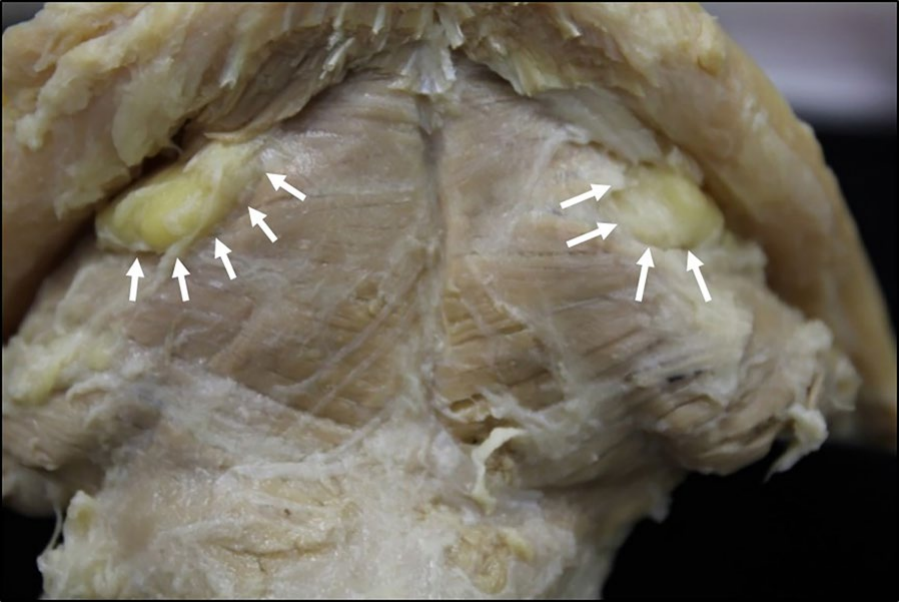
Mylohyoid attachment site viewed from below in which the mylohyoid line and the mandibular symphysis are not continuous (digastric muscle has been cut off at its origin). White arrows: regions in which there is no attachment between muscle and bone

This investigation of the mandibular attachments of the mylohyoid showed that, in all specimens, the mylohyoid originated not only from the mylohyoid line, but also from the lingual surface of the mandibular symphysis below the mental spines (Fig. 4). The terminus of the mylohyoid was at the anterior surface of the center of the hyoid body or the mylohyoid raphe. The anterior mandibular attachment of the mylohyoid (lingual surface of the mandibular symphysis) and the center of the anterior surface of the hyoid body (mandibular symphysis) were connected anteroposteriorly by the mylohyoid raphe, and the mylohyoid raphe constituted the inferior-most line of the mylohyoid (Fig. 5). The posterior muscle fascicles of the mylohyoid ran posteroinferiorly from the bilateral mylohyoid lines and terminated at the anterior surface of the hyoid body on the same side. The central muscle fascicles of the mylohyoid originated from anterior of the mylohyoid line and terminated at the mylohyoid raphe, and the anterior muscle fascicles originated from the anterior mandibular attachment (mandibular symphysis, the lingual surface of the center of the mandibular body), after which they terminated at the mylohyoid raphe. In 50.0% of cases (48/96), continuity between the anterior and central muscle fascicles was lost, and there was a region between the anterior margin of the mylohyoid line and the anterior mandibular attachment (mandibular symphysis) in which there was no mylohyoid attachment (Fig. 6). In all cases, the presence or absence of continuity between the anterior and central muscle fascicles was bilaterally symmetrical.

### Mylohyoid thickness measurements

**Fig. 7:**
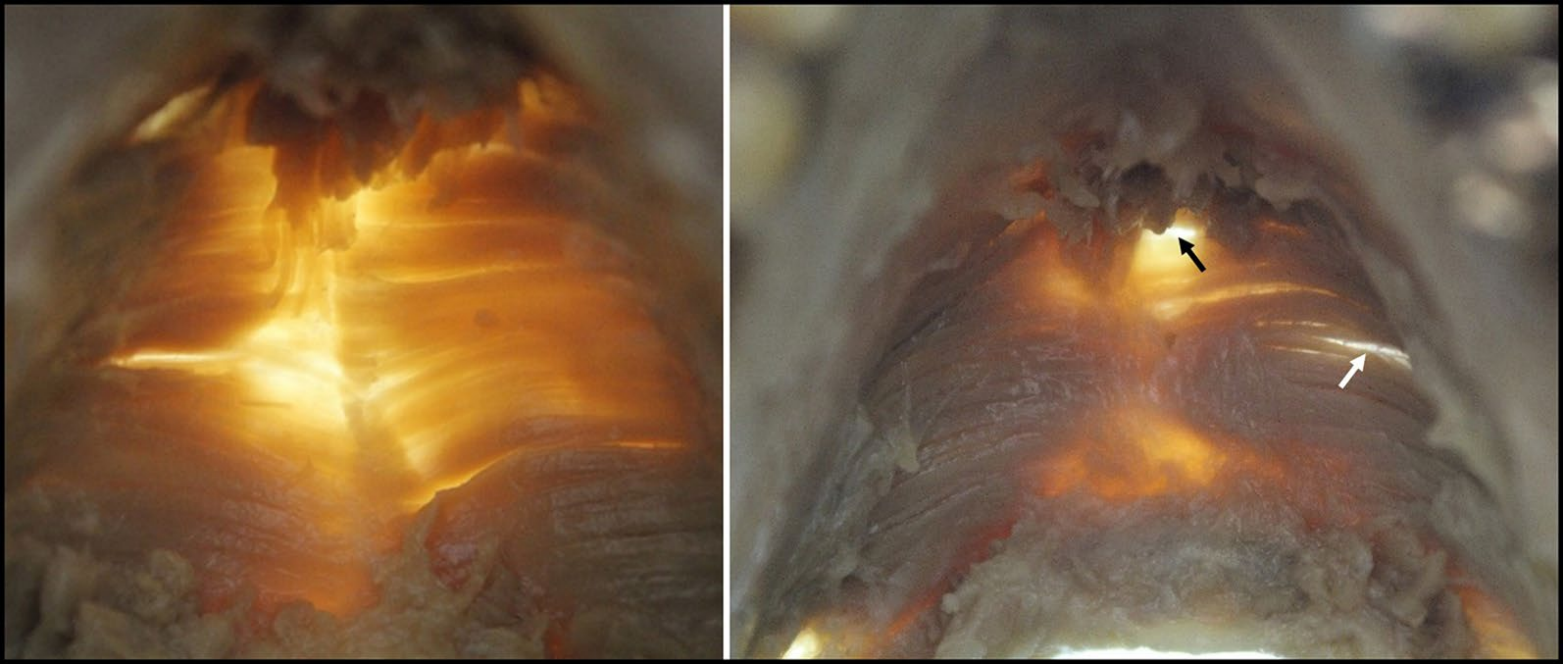
Areas where muscle fibers of mylohyoid are loose (where the light shines through). Left: no penetration. Right: penetration. The anterior part of the mylohyoid is thin, but further posteriorly (to the hyoid bone) it becomes thicker. It is also thinner nearer the mylohyoid raphe, and thicker nearer the mylohyoid line. Black arrow: site of penetration. White arrow: gap between muscle fascicles

Overall, the mylohyoid muscle fibers formed a broad sheet, but its thickness was greatest in the posterior area and decreased further anteriorly, until, in the anterior part, it was less than 1-mm thick (Table 1). In both the central and posterior regions, it was thickest in the area adjoining the mylohyoid line, and it tended to become thinner as it approached the hyoid bone or the mylohyoid raphe (Fig. 7). The fascia of the mylohyoid was present and enclosed the mylohyoid muscle at every point, but it was extremely thin. Sublingual gland herniation penetrating the mylohyoid was present in 23.0% of cases (22/96), of which herniation in the anterior part was present in 54.5% of the total cases of sublingual gland herniation (12/22: 6 right and 6 left), and herniation in the central part was present in 45.5% of the total cases (10/22: 6 right and 4 left), whereas there was no sublingual gland herniation in the posterior part. Sites of penetration other than these were evident in 57.3% of cases (55/96), with penetration most commonly seen in the central part and not present at all in the posterior part. Most of the sites of penetration were at places, where the muscle fascicles were loose between muscle fibers, and consisted solely of loose connective tissue.

**Table 1:** Thickness (mm) of mylohyoid in each region, superior–inferior penetration, and sublingual gland invagination (*n* = 96)

Region	A	B	C		D		E		F	
			C I	C II	D I	D II	E I	E II	F I	F II
Length (mm)	0.67	0.74	1.59	0.94	1.81	1.03	2.45	1.29	2.50	1.44
SD (mm)	0.27	0.27	0.33	0.65	0.65	0.37	0.67	0.41	0.78	0.51
Sublingual gland invagination	6	6	4		6		0		0	
Penetration	9	11	15		20		0		0

### Measurements of the vertical distance from the mandibular plane to the origin

In measurements of the vertical distance from the mandibular plane to the mylohyoid line and the anterior mandibular attachment (lingual surface of the mandibular symphysis), *a* (MFMY) was 11.5 ± 2.0 mm, and *b* (HLMY) was 14.4 ± 2.0 mm (Table 1). For the other distances measured, *c* (the distance from the mental spines to the mandibular plane) was 14.4 ± 2.0 mm, and *d* (the distance from the mandibular symphysis to the mandibular plane) was 5.5 ± 1.6 mm (Table 2).

**Table 2:** Measurements of the vertical position of the mylohyoid attachment in each region (*n* = 96)

	a	b	c	d
Length (mm)	11.52	14.51	14.40	5.52
SD (mm)	2.12	2.05	1.95	1.60

## Discussion

It is well-known that the mylohyoid originates from the mylohyoid line, and this is what is stated in all textbooks. Although *Gray’s Anatomy* describes the anterior mandibular attachment below the mental spines as located at the “symphysis menti,” [16], and Zdilla et al*.*’s paper also describes this site as the “mandibular symphysis” [17], these are very minor mentions in comparison with the mylohyoid line. In the present study, the mandibular symphysis was the origin of the anterior mylohyoid muscle fascicles in all specimens. The mandibular symphysis also played the role of the anterior attachment point of the mylohyoid raphe, and it was connected to the posterior hyoid by the mylohyoid raphe. This showed that the mandibular symphysis thus plays an important role as the location of the anterior mylohyoid attachment to the mandible. However, the anterior muscle fascicles of the mylohyoid were generally very thin, and their attachment tended to be weak.

The present results showed that, in about half of mylohyoids, the mylohyoid line was not continuous with the mandibular symphysis, and that, in the anterior part, there was bilateral communication between the sublingual and submandibular spaces. Whereas sublingual gland herniation was apparent in 23.0% of all cases, superior–inferior penetration caused by loose muscle fascicles was present in 57.3%. The mean thickness of the central muscle fascicles adjacent to the mylohyoid raphe was less than 1.0 mm, whereas in the vicinity of the mylohyoid line, it was approximately 1.7 mm, and there was a tendency for it to be thinner closer to the midline, but there was no evidence at all of a split in the mylohyoid raphe itself. Penetration was most commonly seen in the central muscle fascicles, being present in 36.4% when both sides were included. From the above results, it should be considered that there are numerous routes of superior–inferior communication between the sublingual and submandibular spaces. The present study suggested that the mylohyoid functions only weakly as a septum on the lingual side of the premolar region, and that it may be a vertical route of communication, particularly for bleeding or infection in the sublingual area.

In contrast, the posterior muscle fascicles formed a thick sheet in the vicinity of both the origin and the terminus, with no evidence at all of superior–inferior penetration. The posterior fascicles preserve their thickness up to the posterior margin of the mylohyoid, and further posterior to this posterior margin there was complete communication between the sublingual and submandibular spaces via loose connective tissue. In some cases, the mylohyoid raphe was clearly visible as tendinous tissue, whereas in others, it was apparent as the union of the left and right mylohyoids, but in all specimens, the mylohyoid raphe was always continuous from the mandibular symphysis to the hyoid. The fasciae covering the mylohyoid were generally thin, and at sites where the muscle fascicles were disrupted the submandibular and sublingual spaces were separated only by loose connective tissue. The mylohyoid muscle fascicles can be broadly divided into those that terminate at the hyoid and those that terminate at the mylohyoid raphe, and this structure means that a gap can readily appear at the boundary between these two types of muscle fascicles. In addition, the sublingual glands are often herniated within this gap. This means that sublingual gland herniation and perforation are frequent in the central part of the muscle even at sites, where the muscle fascicles are thick. The mylohyoid is also known as the “oral diaphragm,” and it is generally considered to provide a complete division between the sublingual and submandibular areas above and below. However, what supports its role as a septum is its attachments to the mandible and the hyoid. The above results suggest that, despite the fact that the mylohyoid functions only weakly as a septum, and that routes of communication may be present between the sublingual and submandibular spaces in both its anterior and central parts.

The most frequent complications of implant surgery are infection, implant rejection, implant migration and implant fracture [18–20]. As is known, dental implant migration into the maxillary sinus is a complication sometimes encountered in implant treatment [21, 22]. In addition, Cariati et al. reported a very rare case of displacement of a normally placed implant body into the sublingual space [22]. The authors suggested that the implant displacement was caused by the resorption of the internal jaw cortical. The reason for this is that during the first surgery the implant was severely medially inserted. Consequently, the lack of primary implant stability is the major cause of this complication. If the implant strays into the sublingual space, antibiotics must be administered to prevent Ludwig’s angina and the implant must be removed promptly in the operating room. Furthermore, Choi et al*.* reported that post-oral surgery sublingual hematoma leading to life-threatening airway obstruction requires immediate recognition and prompt management [23]. Most dental implant specialists are alert to the possibility of perforation of the lingual-side cortical bone by the implant as an accidental complication during oral implant insertion in the area from the mandibular incisal region to the premolar region [9, 24–27]. In a cadaver study, Fujita et al*.* also suggested that because the sublingual and submental arteries run through the sublingual space, if an implant perforates the lingual-side cortical bone, it may blindly damage these arteries and cause arterial bleeding, which may result in airway obstruction or other fatal complications [8]. Therefore, we reaffirmed the importance of carefully confirming the placement direction using a surgical guide when performing implant placement in the mandibular anterior region.

## Conclusions

It is clear that although the mylohyoid muscle demarcates the sublingual and submandibular spaces as compartments, it does not play a role in preventing the downward movement of blood and pus according to gravity. In particular, there is a high probability of a fissure in the anterior and middle part of the mylohyoid muscle and a communication routes at the discontinuity between the mylohyoid muscle line and the mandibular symphysis. Therefore, it should be understood that injury to the sublingual or submental arteries in the sublingual space associated with implant placement surgery can result in rapid blood flow from the submandibular space to the lateral pharyngeal space and pretracheal space. In addition, cases of implant bodies displaced into the sublingual space due to resorption of lingual cortical bone have been reported, suggesting that bone resorption should be considered when placing implants in the vicinity of lingual cortical bone during oral implant insertion in the area from the mandibular incisal region to the premolar region.

## Data Availability

The data sets used and analyzed during the current study are available from the corresponding author on reasonable request.

## Abbreviations

LMM: The lower margin of the mandible
MF: Mental foramen
MFMY: Distance between the mylohyoid line and LMM at the vertical line of MF
HLMY: Distance between the mylohyoid line which the half point of the lingula’s notch and MF and LMM
